# OPTIMIZING FUNCTION AND INDEPENDENCE: NATIONAL IMPLEMENTATION OF THREE CLINICAL PROGRAMS WITHIN VA

**DOI:** 10.1093/geroni/igae098.3415

**Published:** 2024-12-31

**Authors:** Jaime Hughes, Caitlin Kappler, Connor Drake, Leah Zullig, Kasey Decosimo, Sara Webb, Susan Hastings

**Affiliations:** Wake Forest University School of Medicine, Winston-Salem, North Carolina, United States; Durham VA Health Care System, Durham, North Carolina, United States; Durham VA Health Care System, Durham, North Carolina, United States; Durham VA Health Care System, Durham, North Carolina, United States; Durham VA Health Care, Durham, North Carolina, United States; Durham VA Health Care, Durham, North Carolina, United States; Durham VA, Durham, North Carolina, United States

## Abstract

Older Veterans aged 65+ account for roughly one-half of Veterans Affairs (VA) Healthcare users. Comprised of more than 170 medical facilities, VA promotes programs to support function and independence for both Veterans and their caregivers. The VA Optimizing Function and Independence Quality Enhancement Research Initiative (Function QUERI) focuses on the national scale-up of three evidence-based programs (EBPs) for older Veterans and their caregivers: STRIDE (hospital-based mobility), Caregivers FIRST (caregiver education and training), and Group PT (group-based rehabilitation for knee osteoarthritis). As part of the Function QUERI program, three individual Type III hybrid effectiveness-implementation trials were conducted to, first, identify baseline organizational characteristics (organizational readiness, implementation climate, and implementation experience) associated with successful program adoption of each EBP; and, second, to evaluate strategies to adopt and sustain each EBP. To date, 142 VA facilities have participated in the implementation of one or more EBPs (STRIDE=67, Caregivers FIRST=142, Group PT N=19). The trials tested a similar implementation approach where all sites received foundational support tools (e.g. program toolkits, documentation templates, technical assistance); and, sites that did not adopt their respective EBP by pre-specified benchmarks received enhanced implementation support via virtual external facilitation (STRIDE= 13, Caregivers FIRST=13, Group PT =9). Barriers to program adoption were similar across EBPs (e.g., competing demands, staffing challenges, coordination between disciplines, poor leadership support). Offered as part of both foundational tools and enhanced support activities, implementation strategies (e.g., adaptation, engage and train stakeholders, monitor and evaluate feedback) were used to address adoption barriers across all EBPs.

